# Spontaneous Erosion of a 15-Year-Old Retained Inflatable Penile Prosthesis Reservoir Into the Bladder: A Case Report and Literature Review

**DOI:** 10.7759/cureus.107422

**Published:** 2026-04-20

**Authors:** Alaa Hamada, Lillian Lai, David Staskin, Shaheen Alanee

**Affiliations:** 1 Urology, John D. Dingell VA Medical Center, Detroit, USA; 2 Urology, Wayne State University School of Medicine, Detroit, USA; 3 Urology, Tufts University School of Medicine, Boston, USA; 4 Urology, Michigan State University, East Lansing, USA

**Keywords:** bladder erosion, penile implant complication, penile implants, reservoir, salvage procedure

## Abstract

In the absence of infection, the reservoir of a malfunctioning three-piece inflatable penile prosthesis (IPP) is often left in place during replacement. Experts believe that the risks associated with reservoir explantation outweigh the risks of a drain-and-retain approach. However, there is no clear consensus on monitoring. The objective of this case report is to highlight the need for vigilance regarding long-term potential complications associated with retained reservoirs, as this report describes a retained reservoir eroding spontaneously into the bladder 15 years post-placement in a 69-year-old man. The patient had been reporting gross hematuria, recurrent UTIs, and worsened lower urinary tract symptoms (LUTS). CT imaging, followed by cystoscopy, demonstrated the decommissioned reservoir in the bladder. The patient then underwent exploratory laparotomy and bladder exploration to remove the eroding reservoir and replace the functional three-piece IPP with a malleable one. By reviewing the available literature, we found three clinical scenarios using variable clinical approaches to deal with bladder erosion by the reservoir, whether functional or decommissioned. In conclusion, patients with retained IPP reservoirs are at potential risk for complications. Prosthesis erosion may appear in the differential diagnosis when patients with a history of IPP placement present with hematuria or LUTS. Long-term investigation is needed to identify patients at high risk of complications and to elucidate proper monitoring and best surgical practices to manage these complications.

## Introduction

Erectile dysfunction (ED) affects approximately one in five men in the United States. Since the first implantation of the inflatable penile prosthesis (IPP) in 1973, it has been a well-established treatment for men with refractory ED [1]. Owing to iterative improvements in device design and operative technique, the 10-year prosthesis survival rate is as high as 77-86%, with 57% of all reoperations attributable to device malfunction [2,3]. Given the high patient satisfaction with IPPs, physicians often replace a malfunctioning IPP with a new prosthesis. During replacement surgery for a malfunctioning IPP, the decommissioned reservoir (DR) is often left in place to obviate reservoir removal morbidities (i.e., the drain-and-retain approach) [4,5]. However, clear guidelines for patient selection and monitoring of retained IPP reservoirs are lacking. Multiple case series have reported reservoir erosion into the urinary bladder during initial primary placement or delayed erosion from both functional and decommissioned prostheses. We present a unique case of retained reservoir erosion into the bladder 15 years after implantation. We review and examine case reports on urinary bladder erosion to determine the potential risk factors for such complications.

## Case presentation

A 69-year-old man with a medical history of obesity, hypertension, and obstructive sleep apnea underwent placement of a Coloplast Titan IPP (Coloplast, Minneapolis, MN, USA) for refractory ED in 2009. Physicians placed the reservoir and pump on the right side of the space of Retzius and the right hemiscrotum, respectively. The procedure lasted three hours. The patient later developed an indirect left inguinal hernia that was repaired in 2014 via an open approach using mesh. Ten years after the initial implantation, the patient underwent explantation and reimplantation of another Coloplast Titan IPP because of mechanical failure. According to the urologist on duty, the old reservoir was difficult to remove through the penoscrotal incision, and no counter-incision was made. After prolonged dissection, the surgeon cut the reservoir tubing as proximally as possible, leaving the DR in place. A new reservoir was then placed ectopically in the high submuscular space under the right rectus abdominis to avoid the left side due to mesh placement.

**Figure 1:**
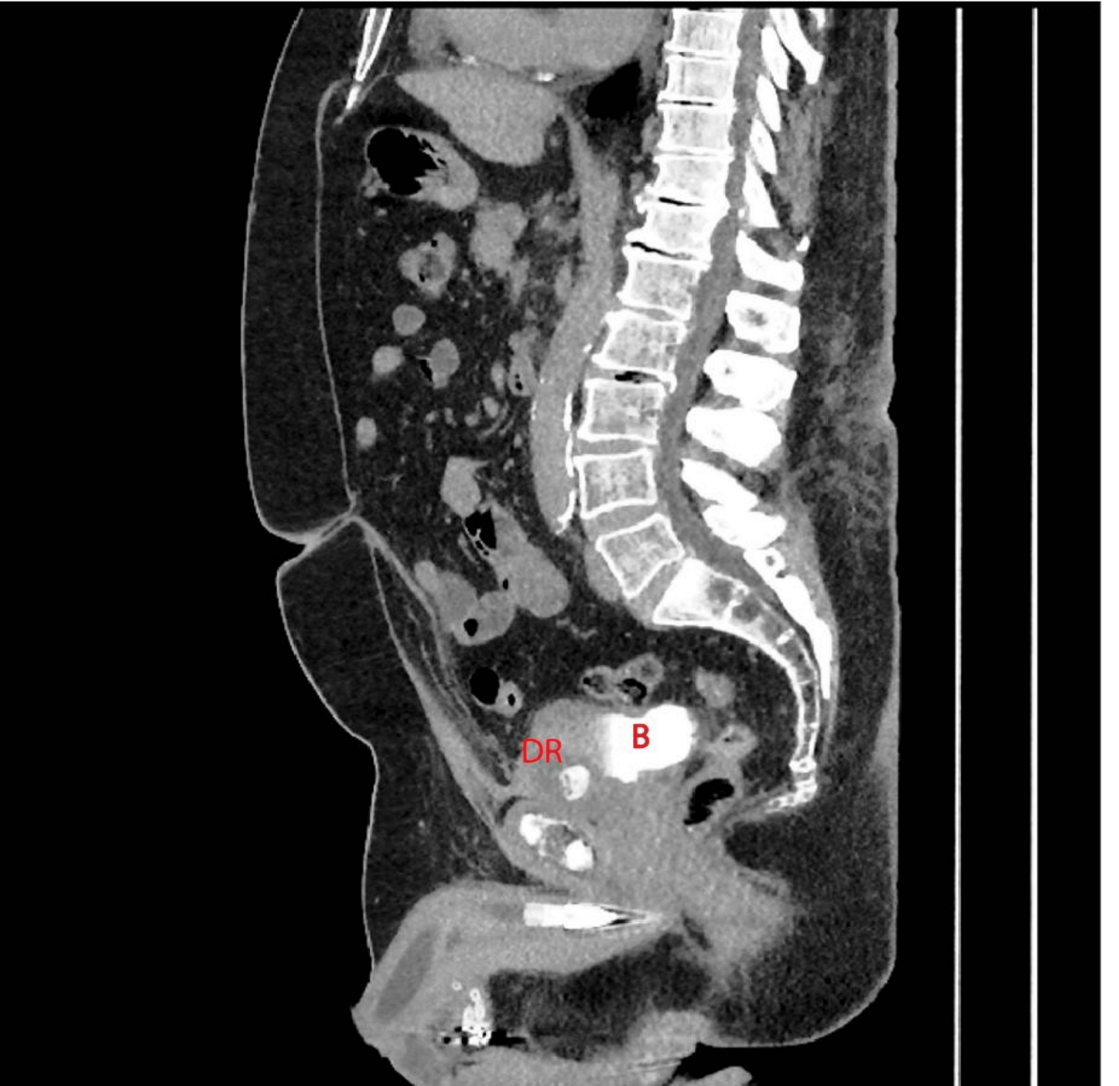
CT urogram longitudinal section showing DR eroding into the bladder B, bladder; DR, decommissioned reservoir

**Figure 2:**
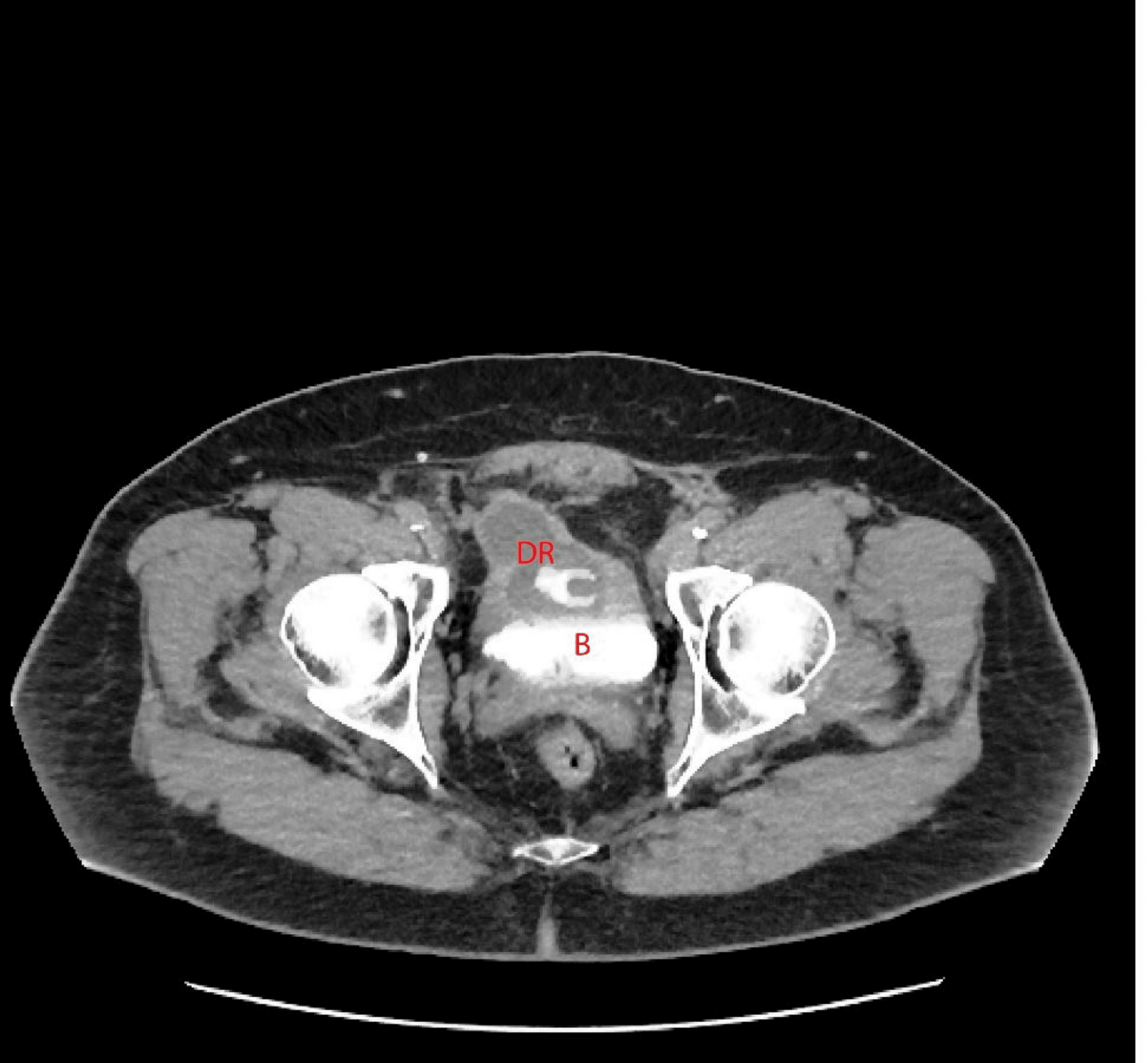
CT urogram transverse section showing DR eroding into the bladder B, bladder; DR, decommissioned reservoir

In 2024, the patient presented to the emergency department with a three-month history of gross hematuria and worsened lower urinary tract symptoms (LUTS). Urinalysis was positive for hematuria and leukocyturia. Urine culture was negative with 1,000 colony-forming units. A CT urogram demonstrated a DR within the bladder with surrounding fat stranding (Figure 1, Figure 2).

**Figure 3:**
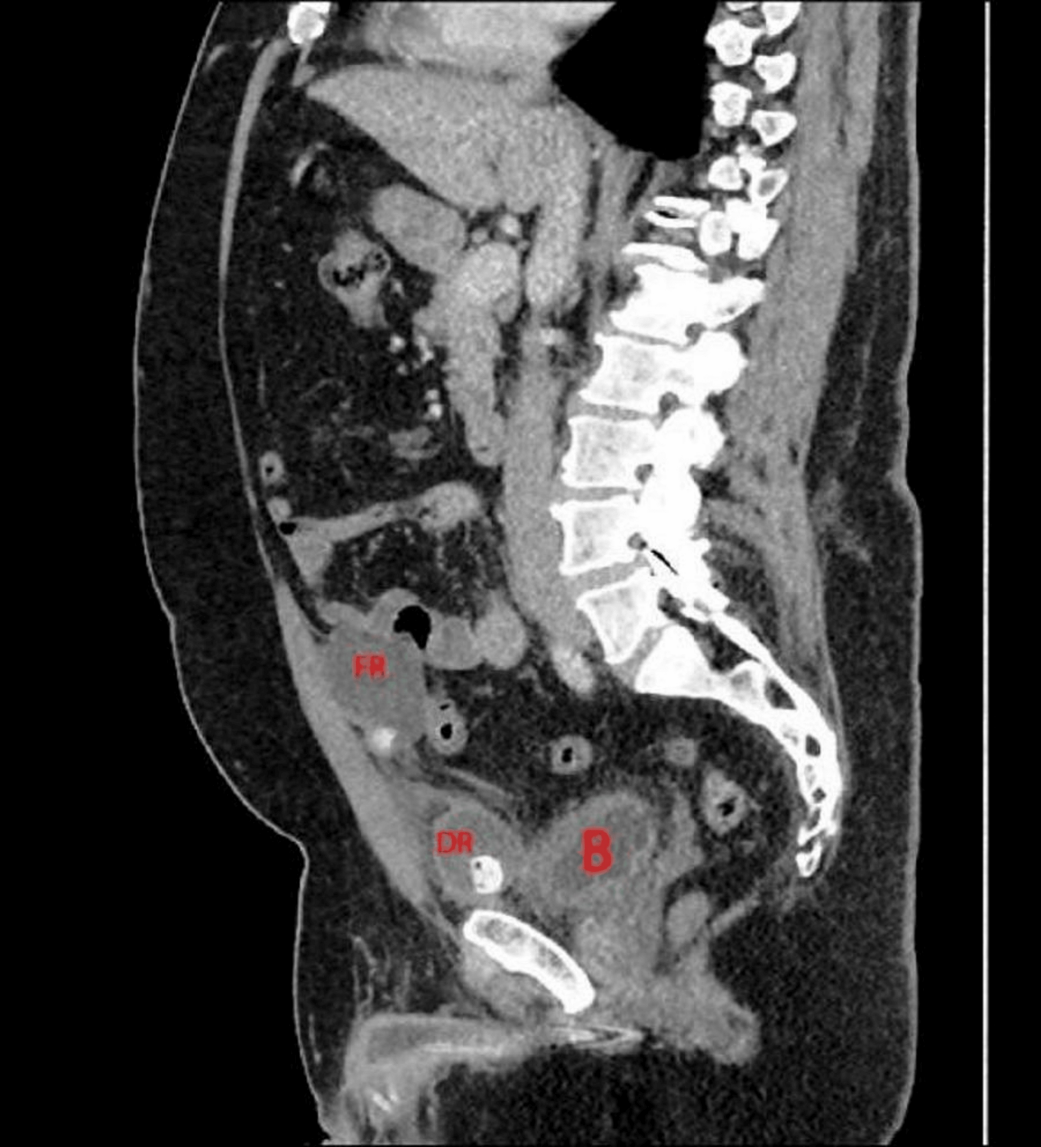
CT urogram showing the DR separated from the bladder The FR is in the submuscular space. B, bladder; DR, decommissioned reservoir; FR, functional reservoir

In reviewing older images, the team found the DR to be close to the bladder. The functional reservoir (FR), which was in the submuscular region, was partly seen intraperitoneally with the surrounding bowel (Figure 3).

**Figure 4:**
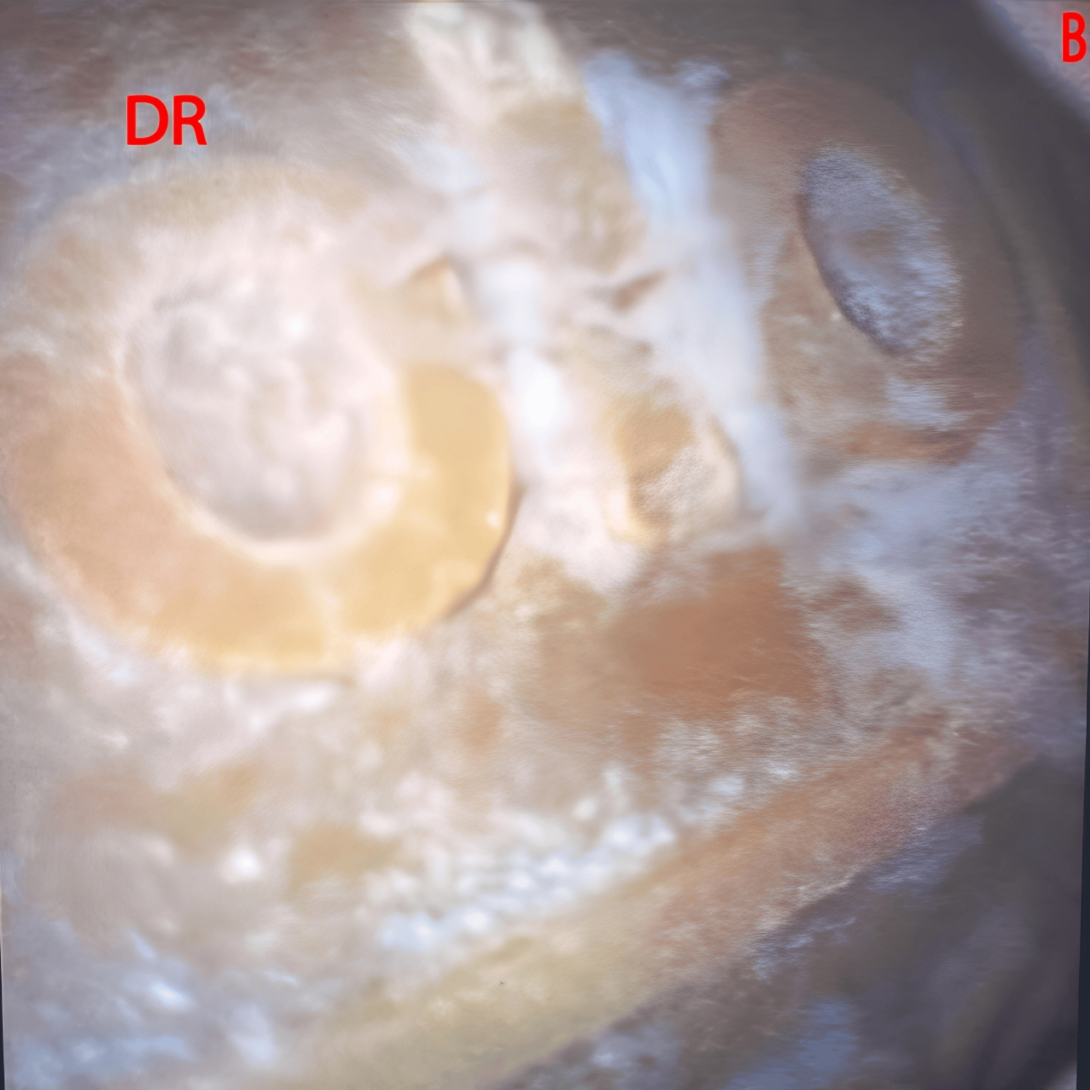
Cystoscopy image showing the DR in the bladder B, bladder; DR, decommissioned reservoir

Emergency cystoscopy revealed a calcified bladder reservoir (Figure 4). The team performed an emergency exploration using a lower midline incision. They then dissected dense scar tissue and omental adhesions to obtain bladder access. Next, they performed a cystostomy and removed the DR. No bladder wall perforation was observed. The cystostomy was then closed, and a Foley catheter was inserted.

**Figure 5:**
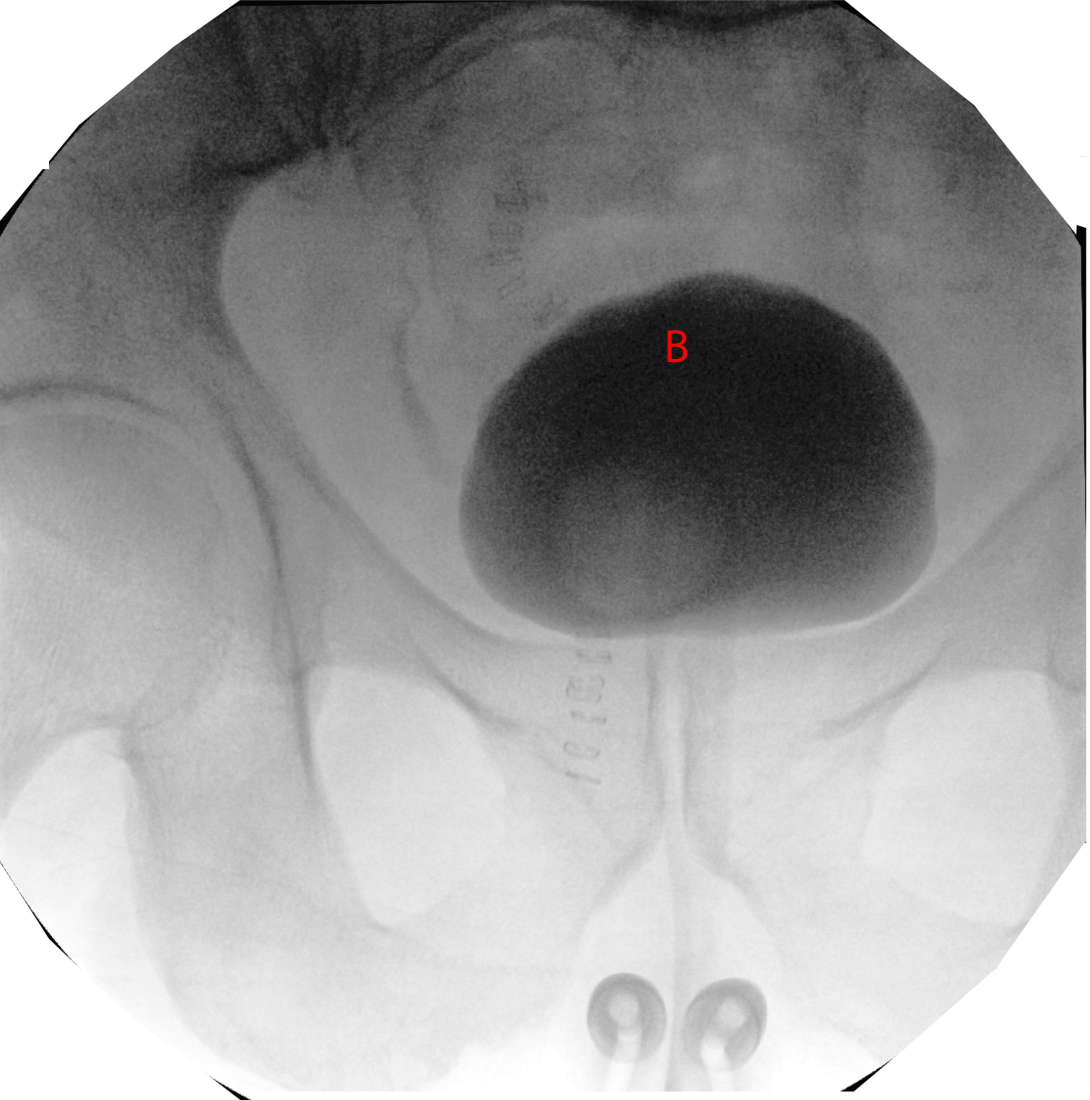
Cystogram image demonstrating no fluid extravasation from the bladder B, bladder

Given the proximity of the FR and the absence of clinical IPP infection and negative urine culture, the team removed the reservoir and replaced it with a new one placed in the high right submuscular space. They then separated the tubing from the midline incision and the two surgical fields by exteriorizing the tubing via the rectus fascia and connecting it to the pump tubing. The IPP cycled well, and the patient returned to the ward for observation with IV vancomycin and piperacillin/tazobactam. Subsequently, the patient developed progressive pain at the site of the new reservoir. On postoperative day 3, the team decided to proceed with IPP explantation and placement of a Coloplast Genesis malleable penile prosthesis due to concerns for prosthesis infection. The patient recovered well thereafter and achieved pain resolution. A cystogram on postoperative day 7 showed no extravasation (Figure 5). During one- and four-month follow-up clinic visits, the patient remained well, with the malleable prosthesis in place and no evidence of infection.

## Discussion

To the best of our knowledge, this is the 10th reported case of delayed erosion of a retained IPP reservoir into the bladder. While leaving the DR in place is a safe approach for many patients, our case highlights that such reservoirs may carry a potential risk of complications. This case is notable because the patient presented 15 years after the IPP was inserted.

Although improvements in device design have increased IPP reliability and longevity, many urologists still encounter malfunctioning IPPs requiring explantation and replacement. In current practice, noninfected DRs are routinely left in place to avoid reservoir removal morbidity. The “drain-and-retain” approach is supported by a five-year experience from a high-volume institution in which 55 reservoirs (artificial urinary sphincter or IPP) were retained [5]. There were no erosions and no significant differences in infection rates between the control and retained reservoir groups. Despite this widespread practice, this approach is not without complications.

Emerging literature has reported complications related to retained IPP reservoirs in urinary tract components such as the ureters, neobladder, ileal conduit, bladder, and prostatic urethra. Reported complications include infection, bladder stones, LUTS, herniation, and erosion, all of which are exceedingly rare [6]. Bladder complications have been reported in at least 27 patients. By examining the reported data, affected men were aged 53-86 years, with a median age of 60 years and a mean age of 61 years. Three clinical scenarios of bladder erosion have been described (Table 1). FRs may be primarily misplaced into the bladder due to iatrogenic factors, as described in 12 cases characterized by early clinical presentation, either discovered intraoperatively or with delayed discovery of up to two months (Table 1) [7-12].

**Table 1:** First clinical scenario of immediate iatrogenic bladder erosion by functional IPP reservoir C/P, clinical presentation; IPP, inflatable penile prosthesis; LUTS, lower urinary tract symptoms; peno-s, penoscrotal; RALP, robotic-assisted laparoscopic prostatectomy; SC, subcutaneous; SOR, space of Retzius

Author	Time to C/P	Age (years)	Prior abdominal/pelvic surgery	Treatment	C/P	Type	Approach	Other risk factors
Kramer et al. (2009) [7]	Few hours	61	No	Salvage removal and replacement of the reservoir	Gross hematuria	-	Peno-S	Salvage case due to tubing breakage
	Few hours	64	No	Salvage removal and replacement of the reservoir in SOR	Gross hematuria	-	Peno-S	Salvage case due to tubing breakage
Izol et al. (2019) [8]	2 months	53	RALP	Exploration, sterilization of the original reservoir, and replacement in the ectopic SC space	Fever, LUTS	Coloplast	Peno-S	-
Baumgarten et al. (2020) [9]	-	-	-	-	-	Coloplast	Peno-S	-
Eldefrawy and Kava (2010) [10]	Immediate	-	-	Exploration and repositioning	-	-	-	-
Levin and Hoeh (2012) [11]	Immediate	56	Bladder diverticulum excision	Exploration and removal of the reservoir and delayed ectopic placement in eight weeks	Gross hematuria	AMS 700	Peno-S	Salvage case due to tubing breakage
Furlow and Goldwasser (1987) [12]	Immediate	37	-	Immediate removal and replacement	-	AMS 700	Peno-S	-
	Immediate	69	-	Immediate removal and replacement	-	-	-	-
	Immediate	70	-	Immediate removal and replacement	-	-	-	-
	-	70	-	Immediate removal and replacement	-	-	-	-
	-	42	-	Immediate removal and replacement	-	-	-	-
	-	70	-	Immediate removal and replacement	-	-	-	-

Second, five other cases have described spontaneous delayed erosion of the FR, with a median time to presentation of 48 months and a mean of 46 months post-placement [13-17]. These delayed erosions shared a potential etiology of multiple revisions of the tubing/pump components, with possible downward traction by the surgeon and/or the patient. Four of the devices in these studies were AMS 700, and one was a Mentor device. While two cases were performed via the infrapubic approach, the remaining cases were performed via the penoscrotal approach (Table 2).

**Table 2:** Second clinical scenario of delayed erosion of the functional penile reservoir into the bladder C/P, clinical presentation; peno-s, penoscrotal

Author	Time to C/P	Age (years)	Prior surgery	Treatment	C/P	Type	Approach	Other risk factors
Park et al. (2005) [13]	7 years	67	No	Removal and salvage of the reservoir in the ectopic site - contralateral	Gross hematuria	Mentor	Infrapubic	Two revisions for pump tubing
Fitch and Roddy (1986) [14]	4 years	54	No	Removal and salvage reservoir placement	Gross hematuria	AMS 700	Peno-S	Tubing rupture correction
Dupont and Hochman (1988) [15]	4 years	46	No	Removal	Bladder stones	AMS 700	Infrapubic	-
Garber and Morris (2013) [16]	7 months	60	-	Removal and delayed replacement in six months	Infection	AMS 700	Peno-S	Multiple revisions ×4
Talib et al. (2022) [17]	3 years	63	No	Removal and salvage reservoir replacement	Gross hematuria	AMS 700	Peno-S	Abdominal trauma

Similar to this case involving bladder erosion by the DR (third clinical scenario, Table 3), at least nine other cases have been reported with a time range between seven and 180 months, with a median and mean of 156 and 122 months, respectively, following the drain-and-retain approach [18-23]. The clinical presentation of bladder erosion included recurrent gross hematuria, UTI, and worsening LUTS. Another case report described the development of a large cyst containing 4 L of purulent drainage surrounding a retained reservoir 15 years after its initial placement [22]. One case involved an AMS 700 device, one involved a Coloplast device, and one involved a Mentor device; no information was provided for the remaining cases. The reported surgical approach was penoscrotal in four cases, with no data on the remaining cases. The common shared risk factor, in addition to prior revision with or without placement of a new FR, was narrowing and scarring of the space of Retzius due to placement of a new reservoir, hernia repair with mesh, infection, colectomy, or radiation.

**Table 3:** Third clinical scenario of bladder erosion by decommissioned IPP reservoir C/P, clinical presentation; EBRT, external beam radiotherapy; FB, foreign body; IPP, inflatable penile prosthesis; LUTS, lower urinary tract symptoms; RALP, robotic-assisted laparoscopic prostatectomy

Author	Time to C/P	Age (years)	Prior abdominal/pelvic surgery	Treatment	C/P	Type	Approach	Other risk factors
Simon et al. (2022) [18]	6 years	73	RALP	Robotic removal of the reservoir	Gross hematuria	-	Penoscrotal	Previous revisions
Munoz and Ellsworth (2000) [19]	4 years	68	-	Removal of the reservoir	Recurrent UTI	AMS	Penoscrotal	-
Jones et al. (2002) [20] (four cases)	7 years (range: 3-15)	-	-	Removal of the reservoir	-	-	-	-
Brusky et al. (2005) [21]	13 years	73	Sigmoid colon resection and EBRT	Removal of the reservoir	Gross hematuria	Mentor	Penoscrotal	Previous revisions and sigmoid colectomy and EBRT
Abboudi et al. (2014) [22]	15 years	68	-	Removal of the reservoir	LUTS and a large cyst with FB abutting the bladder wall	-	-	-
Leach et al. (1984) [23]	2 years	-	-	Removal of the reservoir	-	-	-	-
Our case	15 years	69	Hernia repair with mesh and prior revision	Salvage removal and replacement	Gross hematuria	Coloplast	Penoscrotal	Previous revisions and hernia repair with mesh

The treatment of such erosion is also controversial. For primary iatrogenic placement of the FR, most surgeons have advocated salvage replacement of the reservoir and bladder repair, whereas, for delayed erosion of the FR, some surgeons have contemplated salvage removal of the eroded FR and replacement on the contralateral side, ectopic placement, or delayed salvage replacement. For the third scenario, which involved erosion of the DR, physicians performed removal via open, robotic, or endoscopic/mini-laparotomy approaches. Some patients had an existing malleable or two-piece IPP, whereas one patient had a three-piece functional IPP with a submuscular reservoir. One patient did not undergo reimplantation.

In this case, we removed the eroded reservoir via a lower midline incision; however, because of severe omental adhesions around the partly intraperitoneally placed FR, we performed lysis of adhesions around the reservoir to gain access to the bladder. We then removed the FR and replaced it with a new one and placed it under direct vision in the subrectus space away from the incision. We ensured separation of the two surgical fields and connected the tubing to the pump in a new position as well. However, due to postoperative pain and tenderness over the newly placed reservoir, alongside concerns for infection, we performed an explantation and inserted a new malleable implant. The patient recovered well, with excellent outcomes.

Based on this analysis, removal of eroded FRs or DRs with bladder repair and salvage replacement is an acceptable approach in the absence of clinical infection. Together with our case report, these cases highlight the need for long-term monitoring of patients with retained IPP reservoirs. Prosthesis erosion should remain high in the differential diagnosis when patients with a history of IPP present with hematuria or LUTS.

## Conclusions

Decommissioned IPP reservoirs are routinely left in place during explantation or replacement surgeries to avoid reservoir removal morbidity. However, the drain-and-retain approach is not without risks. This case report highlights the need for long-term vigilance regarding complications. Further investigation is needed to identify patients at high risk of complications and to determine appropriate monitoring strategies.
